# Subretinal fibrosis in neovascular age-related macular degeneration: current concepts, therapeutic avenues, and future perspectives

**DOI:** 10.1007/s00441-021-03514-8

**Published:** 2021-09-03

**Authors:** Louis Tenbrock, Julian Wolf, Stefaniya Boneva, Anja Schlecht, Hansjürgen Agostini, Peter Wieghofer, Günther Schlunck, Clemens Lange

**Affiliations:** 1 Eye Center, Medical Center, Medical Faculty, University of Freiburg, Freiburg, Germany; 2 Institute of Anatomy, Leipzig University, Leipzig, Germany

**Keywords:** Age-related macular degeneration, Degenerative disease, Human retina, Subretinal fibrosis, scar formation, choroidal neovascularization, macular neovascularization

## Abstract

Age-related macular degeneration (AMD) is a progressive, degenerative disease of the human retina which in its most aggressive form is associated with the formation of macular neovascularization (MNV) and subretinal fibrosis leading to irreversible blindness. MNVs contain blood vessels as well as infiltrating immune cells, myofibroblasts, and excessive amounts of extracellular matrix proteins such as collagens, fibronectin, and laminin which disrupts retinal function and triggers neurodegeneration. In the mammalian retina, damaged neurons cannot be replaced by tissue regeneration, and subretinal MNV and fibrosis persist and thus fuel degeneration and visual loss. This review provides an overview of subretinal fibrosis in neovascular AMD, by summarizing its clinical manifestations, exploring the current understanding of the underlying cellular and molecular mechanisms and discussing potential therapeutic approaches to inhibit subretinal fibrosis in the future.

## Introduction

**Fig. 1:**
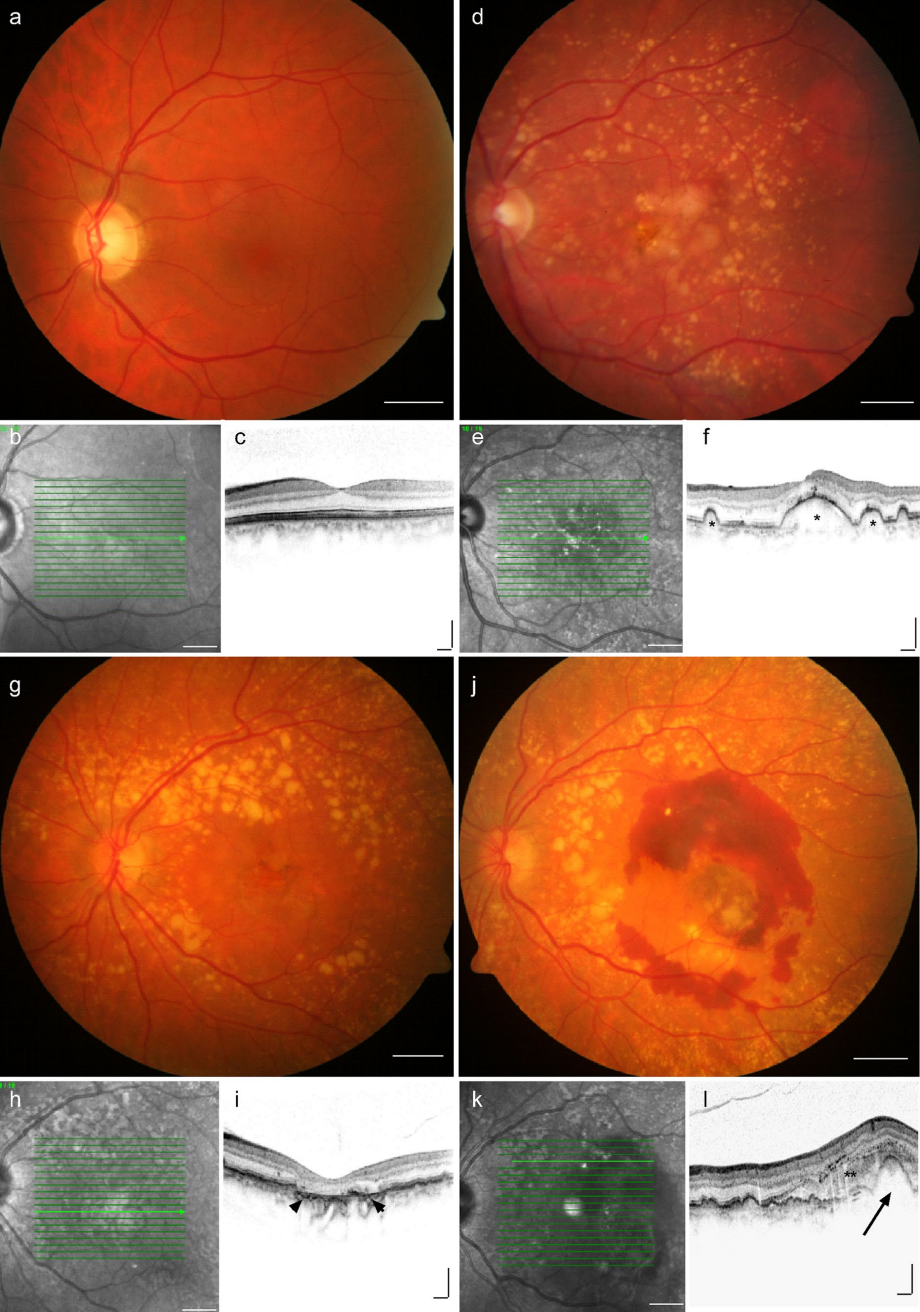
Clinical features of late stages of age-related macular degeneration. Funduscopy, infrared (IR, 830 nm), and optical coherence tomography (OCT) images of patients with a normal fundus (**a**–**c**), intermediate AMD (**d**–**f**), late stage non-exudative AMD (**g**–**i**, geographic atrophy, GA), and neovascular, exudative AMD (**j**–**l**). While healthy individuals reveal a homogenous macular appearance with a foveal dip in OCT (**a**–**c**), patients with intermediate AMD display yellowish deposits, called drusen (**d**, asterisks in **f**) and pigment epithelium alterations. In GA sharply circumscribed areas of RPE atrophy associated with scattered pre-existing drusen can be observed by funduscopy, which are associated with loss of retinal pigment epithelium (RPE) cells (**g**, arrow heads in **i**) and morphologic alterations of the outer retina in the corresponding OCT scan. In nAMD (**j**–**l**), subretinal exudation and haemorrhages are seen on fundus photography, often accompanied by hyporeflective RPE detachment (arrow in l), subretinal fluid, and subretinal hyperreflective material (SHRM, double asterisk) on the corresponding OCT scan (**l**). Scale bar **a**, **b**, **d**, **e**, **g**, **h**, **j**, **k** = 2 mm; **c**, **f**, **i**, **l** = 200 µm

The human macula is a highly specialized region of the central retina and indispensable for high-resolution and colour vision. Histologically, the retina consists of an inner, neurosensory part containing the light-sensitive photoreceptor cells, neuronal and glial cells, and an outer part formed by the retinal pigment epithelium (RPE). The RPE is a monolayer of pigmented cells which forms the outer blood-retinal barrier, is critically involved in the visual process, and separated from the underlying nourishing choriocapillaris by a membrane of collagenous and elastic fibres, called Bruch’s membrane. The integrity of photoreceptors, RPE, Bruch’s membrane, and choriocapillaris as a functional unit is crucial for proper human vision and to a variable extent impaired in age-related macular degeneration (AMD) (Wang et al. 2019). AMD is a progressive, degenerative disease of the human macula and one of the leading causes of blindness among the elderly population in the industrialized world (Flaxman et al. 2017). It is estimated that approximately 288 million people worldwide will be diagnosed with AMD by 2040, highlighting the significant and ever-growing global health burden of the disease (Wong et al. 2014). The exact pathophysiology of AMD is still not completely understood. Clinical features of the early and intermediate stages of AMD include the presence of drusen, which are extracellular deposits of lipids, proteins, and cellular debris that accumulate between the RPE and Bruch’s membrane. While vision can be maintained in early and intermediate stages of AMD, most severe vision loss occurs in late stages of AMD which are classified into two distinctive types: a “dry,” atrophic form and a “wet,” neovascular form of AMD (nAMD; Fig. 1). The former is characterized by map-like zones of cell loss (geographic atrophy) affecting photoreceptors, RPE, and choriocapillaris; the latter by disruption of Bruch’s membrane and formation of macular neovascularization (MNV, previously called choroidal neovascularization, CNV). Three MNV subtypes are distinguished according to the location of the developing blood vessels. Type 1 MNV refers to choroidal blood vessels expanding below the RPE (formerly called "occult CNV"), whereas type 2 MNV is characterized by proliferating choroidal blood vessels breaching Bruch’s membrane and the RPE monolayer to spread in the subretinal space (formerly called "classic CNV"). Type 3 MNV (formerly called Retinal Angiomatous Proliferation, RAP) originate from the retinal vasculature and progress posteriorly into the subretinal space (Spaide et al. 2020). In all cases, the newly built irregular blood vessels are not tightly sealed and are prone to cause leakage and haemorrhage. This disruptive and inflammatory process frequently releases stromal and immune cells which trigger transition of the neovascular endothelial bundle to a fibrovascular membrane. While  fibrosis of macular neovascularization can be beneficial by limiting exudation, excessive fibrosis can culminate in subretinal scarring with irreversible destruction of photoreceptors, RPE cells, and choroidal blood vessels and can thus be responsible for the vast majority of severe visual loss in patients with nAMD (Ferris et al. 1984). To date, there is no therapy for patients with the atrophic form of AMD  . MNV formation in neovascular AMD, however, can be suppressed by intravitreal application of antibodies against the vascular endothelial growth factor (VEGF) and their introduction has significantly advanced the management of nAMD. Nevertheless, significant visual loss (i.e. more than 15 ETDRS letters) occurs in about one in four nAMD patients over an observation period of 10 years despite continuous anti-VEGF treatment (Chandra et al. 2020). This phenomenon has often been attributed to the formation of subretinal fibrosis which occurs in half of the patients with nAMD following anti-VEGF therapy (Daniel et al. 2014). The pathogenetic mechanisms of subretinal fibrosis, however, are poorly understood, and to date, there is no therapy that promotes CNV inactivation while preventing excessive subretinal fibrosis. In this review, we discuss the clinical manifestations of subretinal fibrosis in nAMD, summarize our current understanding of the cellular and molecular mechanisms of subretinal fibrosis in nAMD and provide an overview of potential therapeutic approaches to inhibit subretinal fibrosis.

## Clinical implications of subretinal fibrosis in neovascular AMD

Subretinal fibrosis is the most common natural sequela of MNV and causes damage to the photoreceptors, RPE, and choriocapillaris, resulting in irreversible loss of central vision (Bressler 1988; Wong et al. 2008). Cheung et al. reported an increase of subretinal fibrosis in patients with treatment-naive nAMD from 13.0 to 37.8% within 1 year (Cheung et al. 2019). Histopathological studies show that the severity of photoreceptor damage is proportional to the size of subretinal fibrosis in AMD eyes (Green and Enger 1993), supporting the clinical observation that subretinal fibrosis is the most important predictor of final visual acuity (Cheung et al. 2019). The risk of eyes with nAMD to develop subretinal fibrosis despite ongoing anti-VEGF therapy is reported to be 45% by 2 years (Daniel et al. 2014) and 41% by 10 years (Teo et al. 2020). This is of particular concern because eyes with subfoveal fibrotic scarring after anti-VEGF treatment have the worst prognosis in terms of visual acuity after anti-VEGF treatment (Daniel et al. 2014). Therefore, alternative therapeutic strategies are needed to circumvent the dilemma of inhibiting vessel growth but risking the promotion of scar formation at the same time. Clinically, subretinal fibrosis can be evaluated by funduscopy, fluorescein angiography, and polarization-sensitive optical coherence tomography (PS-OCT) and categorized into fibrotic and non-fibrotic scars (Roberts et al. 2019). While fibrotic scars are characterized as raised clusters of white or yellowish tissue that are well-defined in shape and appear solid on funduscopy, non-fibrotic scars are typically flat, unpigmented lesions with varying amounts of peripheral dark pigmentation. Fibrotic and non-fibrotic scars can both develop with ongoing anti-VEGF therapy, occurring in 24.7% and 20.6% of cases with nAMD after 2 years of therapy, respectively (Daniel et al. 2014). However, it is unclear whether these clinical subtypes of subretinal fibrosis represent pathophysiologically distinct entities or converging disease stages.

**Fig. 2:**
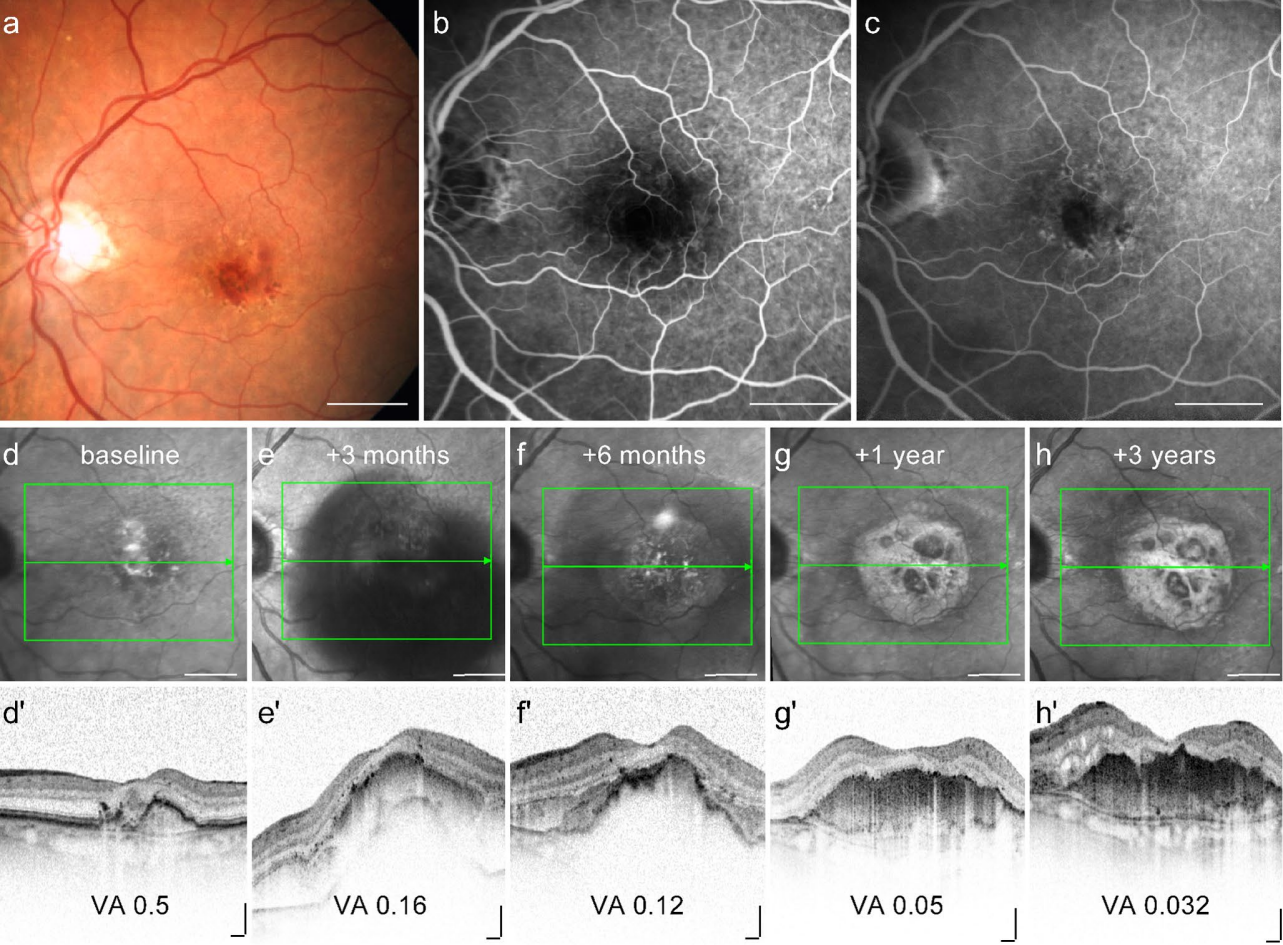
Multimodal imaging of a patient with neovascular AMD showing evidence for macular neovascularisation and submacular fibrosis. Funduscopy (**a**) and fluorescein angiography (**b** early phase, **c** late phase) at baseline indicate risk factors for the development of subretinal fibrosis, including blocked fluorescence. Timecourse of infrared-free (**d−h**) and OCT scans (**d′**–**h′**) of the macula showing a massive subretinal and sub-RPE bleeding at 3 months, followed by a significant formation of subretinal fibrosis and loss of vision to 0.032 after 3 years despite 13 injections of anti-VEGF-treatment. VA = visual acuity (decimal). Scale bar a–h = 2 mm; d′–h′ = 200 µm

There are a number of risk factors for the occurrence of subretinal fibrosis. In general, eyes are more likely to develop subretinal fibrosis during the course of anti-VEGF treatment if they have type 2 MNV (classic CNV), blocked fluorescence on fluorescein angiography (FA) as an indication of bleeding, large basal lesions, increased retinal thickness, foveal subretinal fluid, and subretinal hyperreflective material (SHRM) under the foveal centre at baseline (Fig. 2) (Bloch et al. 2013; Daniel et al. 2018; Teo et al. 2020). It has been proposed that MNV type 2, which penetrate the RPE layer and grow in the subretinal space, are more likely to contain damaged and scattered RPE and thus be more likely to progress to fibrosis than type 1 MNV, which are usually confined to the space beneath the RPE (Ishikawa et al. 2016). Furthermore, several studies suggest that intra-retinal or subretinal haemorrhage, which can result in blocked fluorescence on FA (Fig. 1), is associated with an increased risk of fibrovascular scarring (Scupola et al. 1999; Daniel et al. 2018; Teo et al. 2020) suggesting that cellular and molecular components of the blood can exaggerate scar formation. The detection of SHRM by optical coherence tomography (OCT; Fig. 1) correlates with the formation of a retinal scar and is considered a diagnostic biomarker for fibrosis in nAMD (Casalino et al. 2020). The exact molecular and cellular composition of SHRM is unknown, but it is assumed to be a mixture of fibrovascular tissue, haemorrhage, lipids, fibrin, and immune cells, all of which show similar reflectivity on OCT. Also, a longer interval between diagnosis and treatment (Bloch et al. 2013) and persistent cystoid changes are associated with an even higher risk of developing fibrosis compared to persistent diffuse subretinal fluid (Gianniou et al. 2015). Subretinal fibrosis in patients with nAMD is associated with higher plasma levels of C3a, C4a, and C5a (Lechner et al. 2016); lower serum 25-hydroxyvitamin D concentrations (Singh et al. 2013; Kim and Park 2018); and a higher percentage of circulating CD4 + T-cells as compared to nAMD patients without subretinal fibrosis (Lechner et al. 2015). Interestingly, the incidence of scar formation is relatively low in type 3 MNV lesions which originate from the retinal vasculature and may therefore be more confined to the neurosensory retina without affecting the RPE to the same degree as type 1 and type 2 MNV (Chang et al. 2016; Kim et al. 2018). To date, no genetic and epigentic alterations have been found which predispose an individual to the formation of subretinal scarring. In particular, no association was found between subretinal fibrosis and the common single nucleotide polymorphisms (SNPs, complement factor H, age-related maculopathy susceptibility 2, complement component 3, and toll-like receptor 3) which are known to be strongly associated with the development of AMD (Daniel et al. 2018).

## Basic mechanisms of macular fibrosis

Formation of subretinal fibrosis is based on a still not totally understood interplay of multiple cellular and molecular components. Similar to other fibrotic diseases, subretinal fibrosis in nAMD is the consequence of chronic tissue injury (Friedlander 2007; Wynn 2007) and consists of partially overlapping but functionally distinct temporal phases including cell death and inflammation, cell proliferation and tissue replacement, extracellular matrix (ECM) degradation, and tissue remodelling. A crucial initial prerequisite for the development of MNV is the disruption of Bruch’s membrane, which can occur as a result of inflammatory and degenerative processes of AMD. Subsequently, MNV arise from the proliferation of choroidal endothelial cells, which can eventually penetrate Bruch’s membrane into the subretinal space. These newly formed and leaky vessels contribute to the chronic damage of the surrounding tissue and lead to an environment with an abundance of inflammatory mediators. The following recruitment, activation, and proliferation of different cell types, such as immune cells and myofibroblasts, culminates in excessive ECM deposition and remodelling, which is a major feature of fibrotic healing (Wynn 2007). Eventually, the neovascular lesion may progress into a fibrovascular complex and eventually macular fibrosis, a process known as angiofibrotic switch (Roberts et al. 2019). During the course of the angiofibrotic switch, apoptosis of retinal pigment epithelial cells, endothelial cells, and occasionally macrophages occurs, leading to a dominance of the fibrotic component within the lesion. In this context, selective apoptosis of VEGF-producing cells, such as transdifferentiated RPE, appears to further modulate MNV regression (Hinton et al. 1998a). It is important to note that although fibrosis and the resulting inactivation of pathological CNV may be a desirable process, it can often lead to neuroretinal damage due to the excessive scar response that accompanies it.

### Cellular composition of neovascular fibrosis in nAMD

**Fig. 3:**
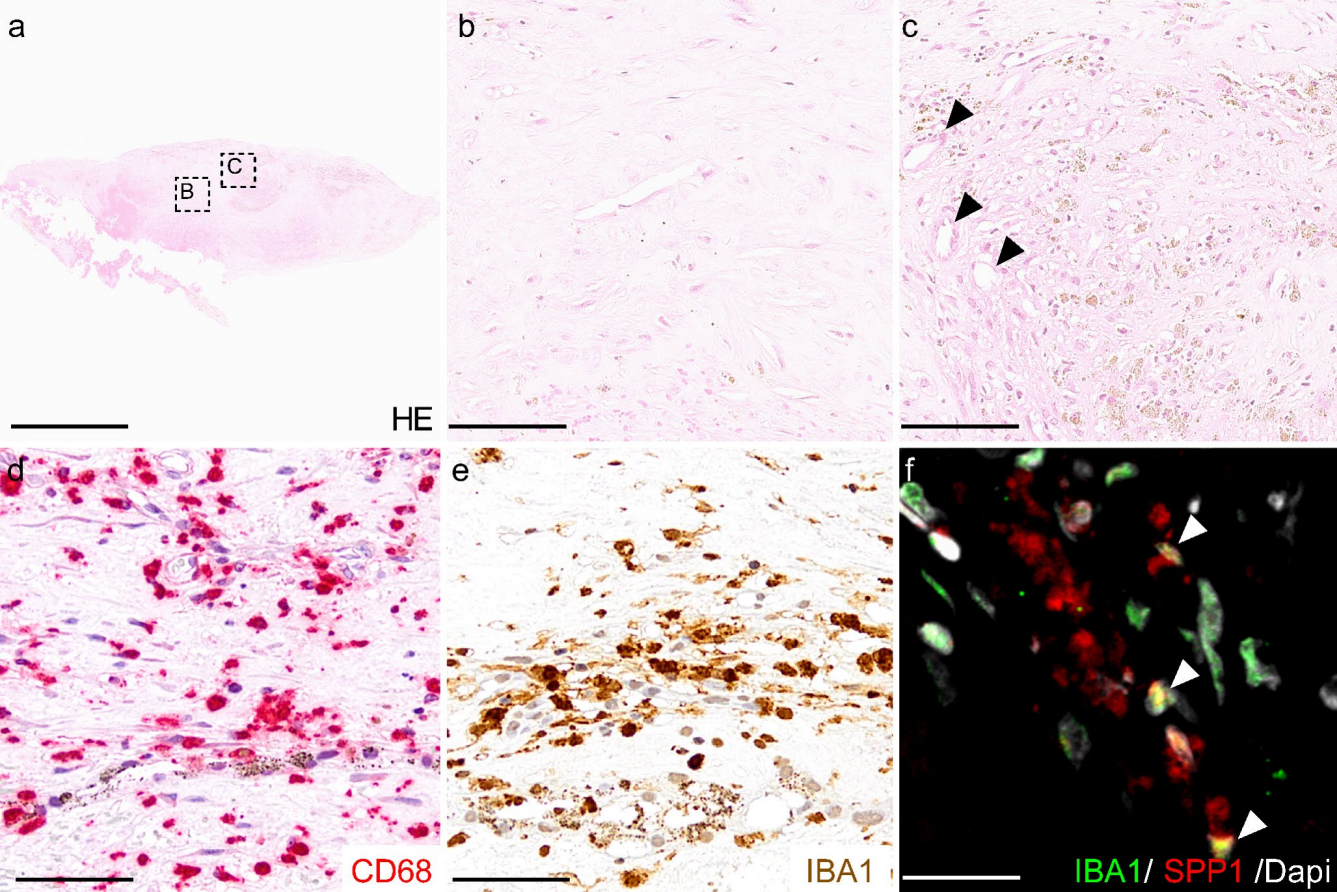
Cellular composition of a human CNV membrane. **a**–**c** Hematoxylin and eosin staining of a surgically excised human fibrovascular membrane showing an overview of the resected tissue (**a**), a fibrotic area with low cell density (**b**) next to an area with increased cell density, and several vessels (some indicated by arrow heads, **c**). The cellular components of a human CNV membrane include CD68 + (**d**) and IBA1 + (**e**, **f**) macrophages/microglia as well as vascular endothelial cells, pericytes, fibroblast-like cells, and myofibroblasts (not shown). Iba1 positive microglia and macrophages contribute to ECM formation by expressing e.g. SPP1 (arrow heads, **f**). Scale bar: **a** = 1 mm; **b**, **c** = 75 μm; **d**, **e** = 50 μm; **f** = 20 μm

**Fig. 4:**
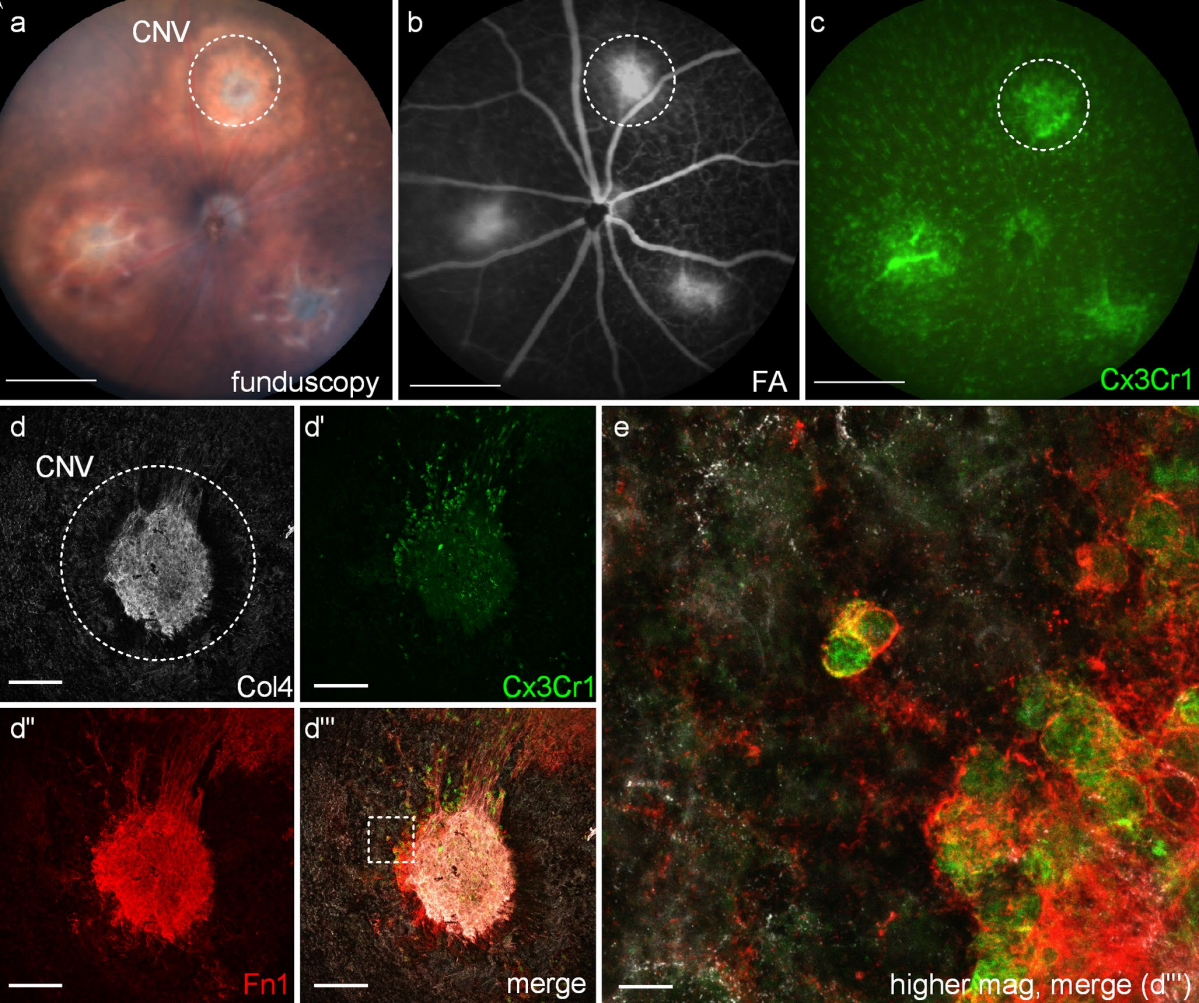
Cellular composition of experimental murine CNV/scar membranes. (**a**–**c**) In vivo imaging of the murine fundus 14 days following laser application (**a**) and the corresponding fluorescein angiography (FA, **b**) showing leakage at sites of CNV membranes (circle) and an accumulation of *Cx3cr1*-GFP + myeloid cells at sites of CNV (**c**). **D**) Immunohistochemistry staining of an experimental murine CNV lesion showing vascular endothelial cells (Col4, **d**), myeloid cells (*Cx3cr1*-GFP + , **d′**) the ECM component FN-1 (**d″**) and a merged image (**d‴**).**e**) Higher magnification of **D‴** demonstrates that myeloid cells contribute to ECM formation by expressing FN-1. Scale bar: a–d = 100 μm; e = 10 μm

Most of our knowledge of cellular composition of MNV lesions is based on histological studies of surgically removed neovascular membranes obtained at the time before anti-VEGF therapy was available by the meanwhile obsolete surgical treatment of patients with nAMD (Fig. 3). Most recently, these investigations were complemented by RNA sequencing and *in silico* analyses of human CNV specimens, providing additional insight into the cellular composition (Schlecht et al. 2020). Further evidence on the cellular mechanisms underlying CNV formation is based on experimental CNV models. Among them, the laser-induced CNV mouse model, first described by Tobe et al. in 1998 (Tobe et al. 1998), is a widely established animal model for neovascular AMD research which is frequently used to test anti-fibrotic drugs. In this model, targeted laser injury to the RPE and Bruch’s membrane induces an acute inflammatory response, recruitment of immune cells, and the formation of choroidal neovascularization (Fig. 4). Similar to the human situation, a fibrovascular switch occurs in the late stages of this model (> 35 days following laser treatment), leading to an α-smooth muscle actin (α-SMA) positive fibrotic lesion (Ishikawa et al. 2016). Recently, a two-stage laser protocol was described in which a second laser burn is applied to the neovascular lesion 7 days after initial laser treatment, resulting in larger lesions than in the traditional laser-CNV model (Little et al. 2020a). Finally, the laser CNV model can be expanded by subretinal injection of activated macrophages into the subretinal bleb which forms after laser-induced rupture of the Bruch’s membrane. This additional treatment results in more pronounced subretinal scar formation 7 days after the injection composed of a monotonous, low-cell-density lesion that expresses α-SMA and collagens (Jo et al. 2011).

**Fig. 5:**
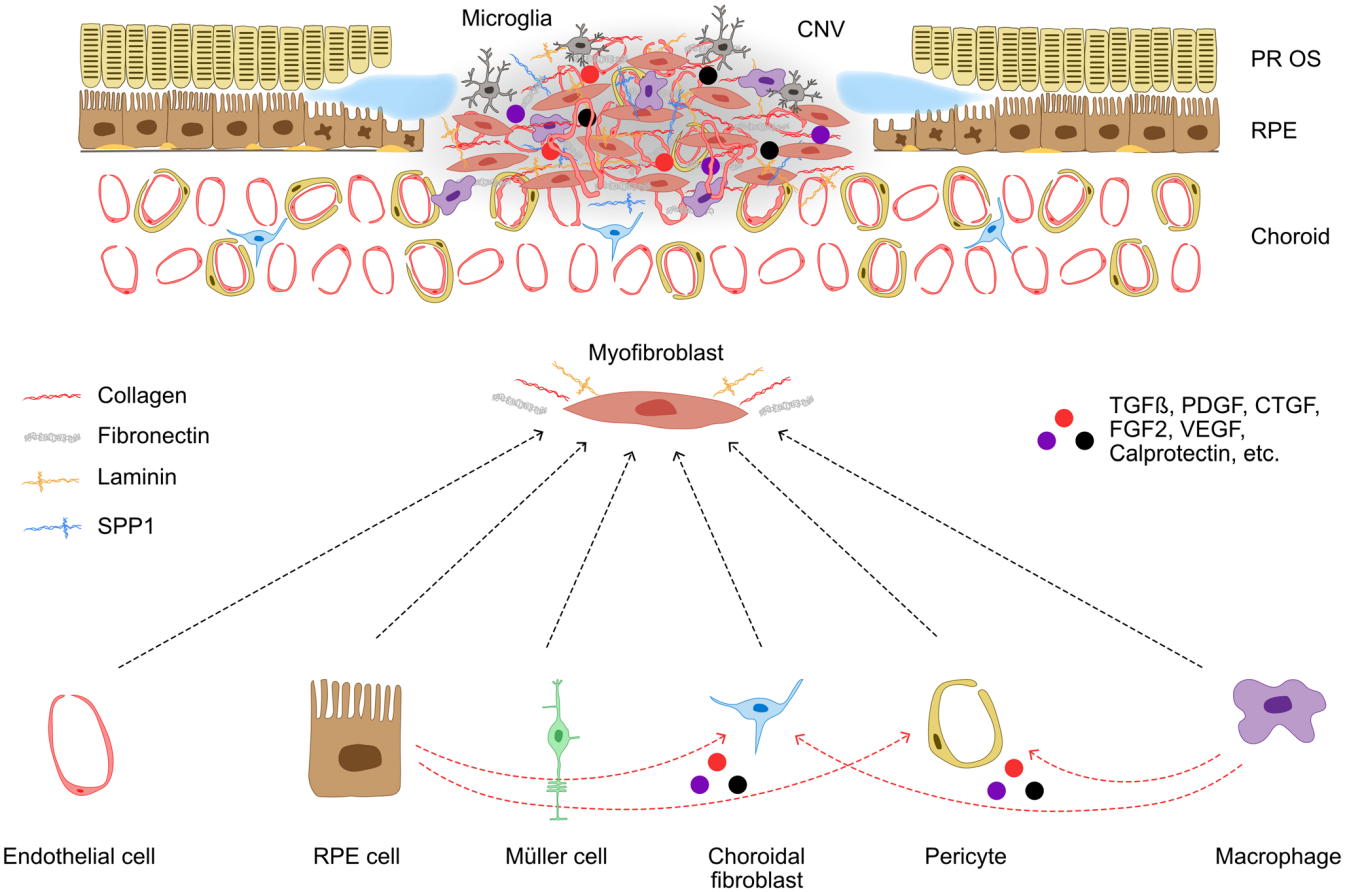
Cellular and molecular interactions in macular scar formation in nAMD. The fibrotic scar is characterized by the deposition of extracellular matrix molecules, such as collagens, laminins, fibronectin and osteopontin (secreted phosphoprotein 1, SPP1), which are otherwise scarcely expressed in the retina and subretinal space. These molecules are mostly generated by myofibroblasts, which are normally absent from the retinal and choroidal parenchyma. The scar stroma cells (myofibroblasts) may originate from endothelial cells, RPE cells, Müller cells, choroidal fibroblast, pericytes, or innate immune cells. Blood‐borne macrophages, resident microglia, and RPE cells contribute to the proliferation and differentiation of stromal cells by producing profibrotic mediators, and they are also involved in eventual fibrous scar resolution. Conversely, stromal cells modulate neuroinflammation by producing cytokines, chemokines, and adhesion molecules. CNV = choroidal neovascularization, PR OS = photoreceptor outer segments, RPE = retinal pigmented epithelium, TGFβ = transforming growth factor β, PDGF = platelet-derived growth factor, CTGF = connective tissue growth factor, FGF2 = connective tissue growth factor, VEGF = vascular endothelial growth factor

Histological studies of human tissue and pre-clinical studies agree that choroidal neovascular membranes are composed of different cell types such as RPE cells, vascular endothelial cells, glial cells, microglia, macrophages, pericytes, fibroblast-like cells, and myofibroblasts (Little et al. 2018). In general, fibrotic lesions in the central nervous system consist of a fibrotic core, which is closely confined by the so-called glial scar. This scar is mainly composed of glial cells, such as astrocytes, confining inflammation to the core of the lesion and protecting surrounding intact neural tissue (Fernández‐Klett and Priller 2014). In the core of the lesion, inflammatory cells, such as resident microglia and blood-derived macrophages (Grossniklaus et al. 1992, 1994; Wieghofer et al. 2021), interact with fibroblasts forming a dense fibrotic scar composed of ECM components such as osteopontin (secreted-phosphorylated protein 1, SPP1), fibronectin, collagen, and laminin (Fig. 5). The development of a fibrous scar is accompanied by an increase in apoptosis and a decrease in cellularity, suggesting that subretinal fibrosis may evolve along with regression of CNV in nAMD (Hinton et al. 1998b).

### Myofibroblasts and the potential source of myofibroblast precursors in subretinal fibrosis

A key element of subretinal fibrosis and expression of ECM are myofibroblasts which typically express α-SMA (Schrimpf and Duffield 2011; Little et al. 2018). Ongoing presence and persistent stimulation of myofibroblasts are associated with excessive ECM deposition which can ultimately result in organ dysfunction (Hinz 2016; Darby et al. 2016). As myofibroblasts are absent in the healthy macula, it is plausible to assume that in nAMD, they develop from myofibroblast precursor cells (Little et al. 2018; Shu et al. 2020). Multiple cell types can contribute to macular fibrosis in nAMD through their activation or trans-differentiation into myofibroblasts. The origin of myofibroblasts in human nAMD is difficult to decipher as respective markers are lost in the transdifferentiation process. Among the potential candidates, RPE cells undergoing epithelial-mesenchymal transition (EMT) are considered a major source of myofibroblasts (Ishikawa et al. 2016; Little et al. 2018; Shu et al. 2020). In the healthy eye, RPE cells exhibit a mature epithelial phenotype and are unable to proliferate due to efficient cell–cell contact inhibition mediated by homotypic adhesion of cadherins to adjacent cells (Yang et al. 2018). Upon loss of cell–cell contact and under the influence of growth factors such as TGFβ, RPE cells lose their epithelial phenotype leading to decreased expression of epithelial markers such as cadherins and zonula occludens 1 (ZO-1) and a disruption of the barrier function of tight junctions. At the same time, an increased expression of mesenchymal markers such as N-cadherin, vimentin, and α-SMA can be observed. Thus, RPE cells can disassociate, gain the ability to proliferate, and undergo EMT contributing to ECM deposition and MNV disease progression (for a detailed review see (Zhou et al. 2020)). In addition, Müller cells, which belong to the glial cells of the retina, can differentiate into myofibroblasts. Porcine Müller cells showed an increased expression of α-SMA under routine cell culture conditions, suggesting that Müller cells can contribute to the myofibroblast pool (Guidry 1996). Transdifferentiation of endothelial cells into myofibroblast-like cells is considered a further origin of myofibroblasts in nAMD (Shu et al. 2020) and was described as endothelial-mesenchymal transition (EndMT). Little et al. (2018) also assumed circulating fibrocytes as myofibroblast precursors as they have been identified in choroidal neovascular membranes (Grossniklaus et al. 1994) and have the potential to differentiate into fibroblasts. This is of particular interest as recent studies in the brain suggest that the majority of fibrotic scarring originates from proliferative CNS fibroblasts that express interferon-gamma pathway genes and collagen1α1 among others (Soderblom et al. 2013; Dorrier et al. 2021). Pericytes have been shown to be myofibroblast precursors in the kidney (Schrimpf and Duffield 2011) and could represent another myofibroblast source in nAMD. Luo et al. identified choroidal pericytes as a source of myofibroblasts in a model of laser-induced CNV and showed that choroidal pericytes play a role in the formation of subretinal fibrosis by expressing ECM components (Luo et al. 2018). Finally, myeloid cells such as macrophages transdifferentiate into myofibroblasts in experimental fibrotic kidney disease (Meng et al. 2016) and express α-SMA under TGFβ induction in vitro (Little et al. 2018). Recent preclinical evidence by Little and colleagues shows that F4/80 + myeloid cells express α-SMA indicating that myeloid cells can undergo TGF-β-induced and C3a-dependent macrophage-to-myofibroblast transition (MMT) at sites of MNV. In line with these findings, approximately 20% of infiltrating Iba-1 + myeloid cells in human MNV were reported to be immunoreactive for α-SMA, suggesting that MMT is present in macular fibrosis secondary to nAMD (Little et al. 2020b) (Fig. 5).

## Immune response

Inflammatory responses, particularly infiltration and activation of cells of the innate immune system, are critically involved in the development and progression of nAMD (Chen and Xu 2015). In particular myeloid cells, such as blood-derived macrophages and resident retinal microglia, can sense exogenous and endogenous danger-associated molecular patterns, migrate to sites of CNV, and drive secondary injury through a vicious neuroinflammatory cycle (Wieghofer et al. 2021). In addition to the functions already mentioned, myeloid cells stimulate proliferation and differentiation of stromal cells by producing pro-inflammatory and pro-fibrotic mediators and contribute to further recruitment of immune cells by secreting chemoattractants. The origin of myeloid cells during CNV formation has long been a subject of debate. Recent evidence suggests that retinal MG constitute the major myeloid cell population in the diseased retina and RPE with lower numbers of recruited monocytes during experimental CNV and GA (Ma et al. 2017; Wieghofer et al. 2021). Depending on the temporal phase of the disease, myeloid cells may exert a dual role with damaging and protective properties. Myeloid cells are involved in immune cell recruitment and secondary tissue damage with resultant scarring in the initial stage. However, they can also adopt an anti-inflammatory phenotype that promotes tissue remodelling and repair through removal of cellular and myelin debris, degradation of scar tissue, and production of neurotrophic factors in later stages (D’Ambrosi and Apolloni 2020). For example, resident microglia contribute to the formation of the gliotic barrier by expressing the matricellullar protein Osteopontin (OPN, gene name *SPP1)* at sites of human and murine CNV (Schlecht et al. 2020, 2021). OPN is expressed by myeloid cells after ischemic or traumatic brain injury and contributes to the formation of the gliotic barrier by binding to α_(v)_β_3_-integrin on astrocytes (Ellison et al. 1998). In the core of the lesion, microglia may contribute to the pro-inflammatory milieu, e.g. by expressing cytokines such as IL-1β, IL-6, and tumour necrosis factor (TNF)-α, which can exacerbate fibrosis in the CNS (Schrimpf and Duffield 2011). Recent evidence suggests that IL-6, expressed by activated microglia, is a crucial mediator promoting subretinal fibrosis and that targeting IL-6 and the corresponding signalling pathway would be an attractive therapeutic approach not only in macular neovascularization, but also in subretinal fibrosis (Sato et al. 2018a). In addition, the presence of activated macrophages, which secrete anti-inflammatory and pro-fibrotic factors such as TGF-β1 and Platelet-derived growth factor (PDGF), has been associated with the accumulation of myofibroblasts and the deposition of fibrillar ECM in the brain (Murray and Wynn 2011). In humans, TGFBR1 expression could be traced back to retinal microglia further corroborating an active role of myeloid cells in this complex interplay of different cell types (Menon et al. 2019). Activated microglia, which secrete pro-inflammatory cytokines, also induce the so-called “A1” reactive astrocytes in neurodegenerative brain diseases such as Alzheimer’s disease (AD), which are no longer able to support neuronal cell survival and differentiation and instead promote neuronal and oligodendrocyte death (Liddelow et al. 2017). On the other hand, macrophages are also crucial for the resolution of fibrosis (Duffield et al. 2005), which in the brain is associated with the expression of IL-13 receptor α2, IL-10 and arginase (Wilson et al. 2007; Pesce et al. 2009). Therefore, a simple categorization of the function of myeloid cells is impossible, as their responses are characterized by deleterious as well as protective properties, which are closely interrelated and mutually dependent. Recent evidence suggests that disease-specific microglia subpopulations with distinct molecular signatures accumulate at sites of experimental murine CNV (Wieghofer et al. 2021). The exact temporo-spatial function of these microglial subpopulations during CNV development is still enigmatic and will be the subject of further studies that may provide the basis for targeted MG modulating therapy in patients with nAMD.

## Growth factors

The aforementioned cells interact and communicate via the secretion of growth factors that control cell recruitment, proliferation, and death in nAMD. Numerous cytokines and chemoattractants contribute to this process. For a detailed overview, the reader is referred to the excellent reviews by (Chen and Xu 2015; Little et al. 2020b).

### TGFβ

Transforming growth factor beta (TGFβ) is a central pro-fibrotic mediator and a master regulator inducing mesenchymal transition of a variety of cells, such as epithelial cells (Xu et al. 2009), endothelial cells (Pardali et al. 2017), and macrophages (Little et al. 2018). TGF-β was found to be expressed in surgically removed CNV tissue (Amin et al. 1994) and in the vitreous of patients with retinal fibrosis (Connor et al. 1989). In the latter, TGF-β2 was found to be more highly expressed than the other TGF-β isoforms and its expression correlated with scar severity (Connor et al. 1989; Pfeffer et al. 1994). TGF-β2 induces epithelial mesenchymal transition of RPE cells predominately by the SMAD3 signalling pathway (Saika et al. 2004) which plays a prominent role in the activation of TGF-β-dependent gene targets, such as plasminogen activator inhibitor-1 (PAI-1) and various collagens, including COL1A1, COL1A2, COL3A1, COL5A2, COL6A1, and COL6A3 (Flanders 2004). Moreover, TGF-β2 signalling can induce TGF-β1, PDGF, and CTGF expression suggesting that TGF-β2 could orchestrate the secondary effects of these other mediators on EMT and fibrosis (Saika et al. 2004). Levels of active TGF-β1 and TGF-β2, but not TGF-β3, were strongly upregulated in mice with subretinal fibrosis in comparison to control mice, and intraperitoneal injection of TGF-β-neutralizing antibodies resulted in the inhibition of subretinal fibrosis (Zhang and Liu 2012). Furthermore, retinoic acid receptor-γ agonist was shown to attenuate TGF-β2-induced EMT in vitro and to suppress experimental subretinal fibrosis in mice (Kimura et al. 2015). Similarly, the inhibition of the transcriptional regulator myocardin-related transcription factor A (MRTFA) leads to reduced expression of EMT markers induced by TGFβ in human RPE cells and is effective in inhibiting subretinal fibrosis development in vivo. Interestingly, MRTFA did not affect CNV formation indicating that it might directly target subretinal fibrosis (Kobayashi et al. 2019). Therefore, inhibition of the TGF-β pathway has been advocated as an additional treatment for neovascular AMD. However, further efforts are necessary to clarify its controversial and cell-dependent involvement in nAMD pathogenesis (Tosi et al. 2018).

#### CTGF

Connective tissue growth factor (CTGF) plays a role in various biological processes and is considered an important regulator of the molecular mechanisms that take place in the process from wound healing to tissue fibrosis (Ramazani et al. 2018). CTGF can be detected in MNV by immunohistochemistry and stained more intense in MNV classified as moderate or extensive fibrosis indicating that CTGF may promote ECM deposition (Watanabe et al. 2005). Preclinical studies summarized by Ramazani et al. indicated that targeting CTGF may serve as a potential therapeutic target to inhibit fibrotic processes (Ramazani et al. 2018). Using the laser-induced CNV-model Daftarian et al. found that inhibition of CTGF resulted in significant reduction of subretinal fibrosis (Daftarian et al. 2019) suggesting a role for CTGF in context of nAMD and fibrosis.

#### FGF

The fibroblast growth factor (FGF) family comprises 23 members with 22 of them found in humans (Yun et al. 2010). FGF2, also known as basic FGF, has been identified in human choroidal neovascular membranes (Frank et al. 1996). FGF2 stimulated TGFβ-induced EMT of RPE cells. The inhibition of FGF2 was associated with decreased CNV and subretinal fibrosis in a mouse laser-induced CNV model (Matsuda et al. 2019).

#### PDGF

The platelet-derived growth factor is a well-established central mediator of vascular homeostasis and pericyte differentiation and a key player in the regulation of fibrotic processes throughout the human body (Andrae et al. 2008). Thus, PDGF represents an attractive target to reduce CNV formation and concomitant subretinal fibrosis (Erber et al. 2004). In preclinical models, anti-PDGF therapy detached pericytes from underlying endothelial cells during ocular neovascularisation (Mitchell et al. 2008), making endothelial cell-lined neovascular tubes highly susceptible to the effects of anti-VEGF therapy (Erber et al. 2004; Jo et al. 2006). Further studies suggest that pericytes and PDGF play a key role in forming a scaffold for endothelial cells during the early stages of experimental CNV formation (Strittmatter et al. 2016) and that PDGF is a potent chemoattractant for immune, RPE, and glial cells (Andrae et al. 2008). In a laser-induced CNV mouse model suppression of PDGF signalling by blocking one of its receptors, the platelet-derived growth factor receptor β (PDGFRβ) resulted in attenuated CNV formation and reduced subretinal fibrosis (Liu et al. 2020) thus paving the way for evaluating anti-PDGF therapy in clinical trials.

### Extracellular matrix

Histological studies showed that choroidal neovascular membranes are composed of ECM components such as collagen, fibronectin, and laminin (Grossniklaus and Green 1998; Grossniklaus et al. 1994; Hinton et al. 1998a, b; Lopez et al. 1996). Recent RNA sequencing analysis of human CNV membranes furthermore revealed increased COL6A1, COL3A1, SPP1, and SPARC expression in human CNV membranes compared to control tissue (Schlecht et al. 2020). Furthermore, periostin, which is a ligand for several integrins and thus supports epithelial cell adhesion and migration, is an important ECM component in human MNV (Yoshida et al. 2019) and has been discussed as a possible anti-fibrotic therapeutic target (Nakama et al. 2015).

### Potential therapeutic targets

To date, there is no anti-fibrotic treatment available to reduce subretinal scarring in patients with nAMD. Clinical trials showed that anti-VEGF monotherapy cannot prevent the development of macular fibrosis (Bloch et al. 2013; Roberts et al. 2019), although it was suggested that timely initiation of therapy may be beneficial in preventing fibrosis (Bloch et al. 2013). This could be explained mainly by its inhibitory effect on angiogenesis and vasopermeability, which reduces the infiltration of immune cells and thus the extent of the inflammatory response. Since the occurrence of fibrosis is the most important predictor of final visual acuity (Cheung et al. 2019), recent efforts have been made to target fibroblast proliferation in nAMD to limit scarring. As discussed above, there is a growing list of molecular mediators and pathways that could be exploited in the development of novel anti-fibrotic drugs. These include cytokine, chemokine and toll-like receptor (TLR) antagonists, angiogenesis inhibitors, TGFβ signalling modifiers, immune cell modulators, or stem/progenitor cell transplantation strategies, to name just a few (see Table 1). In light of the encouraging pre-clinical data on anti-PDGF therapy to suppress fibrosis (see above), recent clinical trials have assessed the efficacy of a combined anti-PDGF and anti-VEGF treatment compared with anti-VEGF monotherapy in patients with nAMD (Jaffe et al. 2016, 2017). The rationale for targeting both molecules was to exploit the anti-fibrotic properties of anti-PDGF and to improve the anti-VEGF efficiency by stripping of pericytes from newly formed vessels thus exposing them to anti-angiogenic therapy.

**Table 1:** Summary of selected, relevant anti-fibrotic drugs for the treatment of experimental subretinal fibrosis. Alphabetical order. No claim to completeness. CNV: choroidal neovascularization, OIR: oxygen induced retinopathy, RPE: retinal pigment epithelium

Therapy	Model	Route of administration	Reference
Ac-EEED (actin blockage)	Mouse, laser CNV	intravitreal	Caballero et al. (2011)
Adrenomedullin	Mouse laser CNV	intravitreal	Tanaka et al. (2020)
Cavtratin (caveolin-1)	Mouse laser CNV	intravitreal	Shimizu et al. (2020)
Connective tissue growth factor antagonism	Rat laser CNV	intravitreal	Daftarian et al. (2019)
Cyclooxygenase 2 antagonism (NS-398)	Mouse laser CNV	intraperitoneal	Zhang et al. (2016)
Doxycyclin	Mouse laser CNV	intraperitoneal	Peng et al. (2018)
Fibroblast growth factor 2 antagonism (RBM-007)	Mouse and rat laser CNV	intravitreal	Matsuda et al. (2019)
Heat shock protein 70	Mouse subretinal fibrosis	intravitreal	Yang et al. (2013)
Interleukin 6 antagonism	Mouse subretinal fibrosis	intravitreal	Sato et al. (2018b)
Interleukin 6 receptor antagonism	Mouse subretinal fibrosis	intravitreal	Sato et al. (2018b)
	Mouse subretinal fibrosis	intraperitoneal	Cui et al. (2014)
Lysyl oxidase and lysyl oxidase-like 2 antagonism	Mouse laser CNV	intraperitoneal	Van Bergen et al. (2015)
Myocardin-related transcription factor A inhibitor	Mouse laser CNV	intravitreal	Kobayashi et al. (2019)
Periostin antagonism	Mouse laser CNV	intravitreal	Nakama et al. (2015)
Phosphatidylinositol-3-kinase inhibitor (3-MA)	Mouse laser CNV	intraperitoneal	Bo et al. (2020)
Platelet Activating Factor Receptor antagonism	Mouse laser CNV	intraperitoneal	Zhang et al. (2013)
Platelet derived growth factor receptor-β antagonism	Mouse laser CNV	intravitreal	Liu et al. (2020)
(Pro) Renin receptor antagonism	Mouse laser CNV	intravitreal	Liu et al. (2019)
Rho kinase inhibitor (AMA0428)	Mouse laser CNV	intravitreal	Hollanders et al. (2015)
Sphingosine-1-phosphate antagonism	Mouse laser CNV	intravitreal	Caballero et al. (2011)
Transforming growth factor beta antagonism	Mouse subretinal fibrosis	intraperitoneal	Zhang and Liu (2012)

In 2017, Jaffe et al. reported that anti-PDGF and anti-VEGF combination therapy was more effective than anti-VEGF monotherapy in improving visual acuity and in limiting the development and progression of fibrosis in a Phase IIb randomized clinical trial (Jaffe et al. 2017). In eyes with visual acuity loss at 24 weeks post treatment, anti-PDGF and anti-VEGF combination therapy was associated with significantly less subretinal fibrosis and less subretinal fibrosis progression (10% and 27%, respectively), compared with eyes receiving anti-VEGF monotherapy alone (51% and 54%, respectively). These encouraging results paved the way for a large phase III clinical trial (NCT01944839), which, however, failed to replicate the success of the phase 2b trial and did not meet the primary endpoint of mean change in visual acuity at 12 months (Hussain and Ciulla 2017). Detailed results of this study, especially on fibrosis formation, have not been published to our knowledge.

## Conclusions

Subretinal fibrosis is the most common natural sequela of macular neovascularization and develops despite successful anti-VEGF therapy. Current evidence suggests that subretinal fibrosis and its ECM components are produced mainly by activated myofibroblasts that transdifferentiate from RPE, pericytes, endothelial, glial, and immune cells, leading to a secondary response involving the innate immune system. The biological function of subretinal fibrotic material deposition most likely is to limit the spread of tissue injury, but the associated prolonged and massive reaction eventually leads to irreversible vision loss and impedes tissue regeneration. While wound healing is to some extent a useful process in nAMD, it would be therapeutically helpful to modulate scarring, resolve scar tissue and thus improve photoreceptor survival by manipulating for example, myofibroblasts derived from stroma and immune cells. Although the targeted inhibition of single factors, such as PDGF, has shown first promising clinical results, the pathophysiology of multiple signalling pathways in scar formation seems to be too complex to achieve relevant clinical success by inhibiting a single factor alone. Nevertheless, an increasing understanding of the involved ECM molecules may lead to the identification of new potent anti-fibrotic therapeutic targets that can be inhibited by intravitreal drug administration. Furthermore, the investigation of ECM components and scarring mechanisms in organisms capable of CNS regeneration, such as the zebrafish, may lead to the identification of pro-repair targets. For example, the Wnt/β-catenin signalling controls the differential regulation of collagen XII within the scar matrix and thus contributes to the pro-regenerative phenotype in zebrafish. These findings imply that the Wnt/β-catenin pathway and Collagen XII may also be targets for extracellular matrix manipulations in neovascular AMD (Wehner et al. 2017). Finally, broader therapeutic strategies aimed at inhibiting mesenchymal transdifferentiation or modulating fibroblasts and active glial and immune cells could limit the associated chronic inflammation and thus effectively reduce fibrosis. However, there are challenging hurdles that need to be overcome before treatment can be translated into clinical practice. Although considerable progress has been made in recent years, our understanding of the molecular and cellular mechanisms needs to be further enhanced. Continued scientific studies on human CNV membranes exploiting single-cell proteomics, single-cell RNA sequencing, and systems biology approaches will be necessary to define the cell types involved and the signalling pathways activated in them. Single-cell RNA sequencing approaches, for example, can reveal the mRNA expression profile of complex and rare cell populations in MNV, uncover regulatory relationships between genes, and track the trajectories of distinct cell lineages in disease progression (Hwang et al. 2018). These studies can be complemented by proteomic studies, e.g. by mass spectrometry, on MNV tissues, which will allow further delineation of the mechanisms involved in the diseases and identification of new biomarkers or drug targets. In addition, systems biology approaches will be valuable to integrate genomic, transcriptomic, proteomic, pharmacological, and clinical data from patients with nAMD into mathematical models that may predict disease onset and progression, identify biomarkers, and eventually unravel disease-causing mechanisms (Handa et al. 2019). However, as human MNV membranes are no longer surgically extracted in clinical practice, these single cell analyses will be difficult to accomplish. Therefore, further efforts should be made to establish novel and optimised animal models that resemble the human situation and the clinical picture of AMD even more closely and may thus be available for the preclinical evaluation of potential future therapeutic approaches. Another difficult obstacle will be the design of effective clinical trials with well-defined clinical endpoints. For this purpose, predictive serum markers, improved imaging techniques or other clinical features that can serve to accurately monitor the course of subretinal fibrosis in a quantitative manner are needed. In addition, the search for genetic factors, such as single nucleotide polymorphisms (SNPs), should continue to determine the individual relative risk of developing fibrosis. The identification of a predictive genetic signature associated with a high risk of developing subretinal fibrosis would be crucial to detect fibrosis processes early and apply an anti-fibrotic therapy specifically tailored to the patient. Since the incidence of AMD and thus the number of patients suffering from subretinal fibrosis continues to increase, there will be a high and increasing need in anti-fibrotic drugs that are both safe and effective.
